# Common symptoms for a rare disease in a girl with sarcoidosis: a case report

**DOI:** 10.1186/s13052-018-0517-6

**Published:** 2018-06-28

**Authors:** Mattia Giovannini, Michele Luzzati, Giovanna Ferrara, Anna Maria Buccoliero, Gabriele Simonini, Maurizio de Martino, Rolando Cimaz, Teresa Giani

**Affiliations:** 1 University of Florence, piazza di San Marco, 4, 50121 Florence, Italy; 2 University of Trieste, via dell’Istria, 65/1, 34100 Trieste, Italy; 3 Anatomic Pathology Unit, Meyer Children’s University Hospital, viale Pieraccini, 24, 50139 Florence, Italy; 4 Anna Meyer Children’s University Hospital, viale Pieraccini, 24, 50139 Florence, Italy

**Keywords:** Paediatric sarcoidosis, Hepato-splenomegaly, Lymphadenopathy, Asthenia, Mycophenolate mofetil

## Abstract

**Background:**

Sarcoidosis in pediatric age is uncommon and challenging diagnosis, because manifestations can be significantly variable and non-specific since it is a multisystem disease, and virtually any organ system may be involved.

**Case presentation:**

In this report, we describe the case of a 12-year-old girl presenting with fatigue and weight loss, with a painless hepato-splenomegaly without additional clinical signs on physical examination. In our patient, once we had ruled out infections, malignancies and granulomatous diseases of childhood, we made diagnosis of sarcoidosis, finding suggestive histological features in two different tissues (liver and lymph nodes) with lung involvement.

**Conclusions:**

Our case points out that pediatricians should consider sarcoidosis in the differential diagnosis in case of systemic symptoms, even in absence of other specific clinical clues, because they represent the most common clinical manifestations on presentation in children, in order to refer promptly the young patient to specialist evaluation.

## Background

Sarcoidosis is a multi-organ granulomatous disease, characterized by non-caseating granulomas.

Two distinct types of sarcoidosis have been described in childhood: the genetic and the classic one (also known as pediatric-onset adult type sarcoidosis). The first one is a monogenic autoinflammatory disease caused by mutations in the *CARD15/NOD2* gene, that includes Blau syndrome and early-onset sarcoidosis, which are the familial and the sporadic forms of the same disease spectrum. Genetic sarcoidosis, which usually develops during the first years of life, is characterized by the clinical triad of recurrent granulomatous uveitis, dermatitis, and symmetric arthritis-tenosynovitis [1, 2].

Classic sarcoidosis is a multifactorial disease, which typically appears in early adulthood [3]. The incidence varies widely throughout the world, from 1 up to 40 cases per 100,000 [4]. With an estimated annual incidence of 2–3 cases per million, sarcoidosis in pediatric age is uncommon and involves mainly pre-adolescents and adolescents [5]. The clinical expression of this disease is protean and depends on the location of the granulomas. Pulmonary involvement is observed in most cases [6], especially in patients with a late onset, and it is frequently associated with extra-pulmonary and constitutional symptoms, while isolated extra-pulmonary disease is very uncommon.

We describe the challenges in considering sarcoidosis as a rare cause of systemic symptoms such as fatigue and weight loss in a young girl, illustrating the importance of considering this diagnosis even in pediatric patients and in absence of respiratory symptoms.

## Case presentation

A 12-year-old girl presented to her general pediatrician complaining of asthenia and weight loss in the previous month. Her past medical and family history were unremarkable. Physical examination revealed a painless hepato-splenomegaly without additional clinical signs. Abdominal ultrasound revealed an inhomogeneous liver appearance, abdominal lymphadenopathy and a hypo-echogenic solid neoformation in front of the celiac artery (35 × 13 mm). The patient was admitted to the Department of Pediatrics of the Meyer Children’s Hospital for further diagnostic investigations.

Physical examination revealed palpable spleen and liver, a right inguinal lymph node of 1 cm and a lymph node in supraclavicular location, without any further objective anomaly. She had no fever. Initial laboratory tests found microcytic iron deficiency anemia (Hb = 9.8 g/dl, MCV = 65.5 ft., Ferritin = 4 ng/ml), with normal erythrocyte sedimentation rate (ESR) and C-reactive protein (CRP), normal total protein and protein electrophoresis, a slight increase of lipase and colic acids with normal amylase. Blood biochemistry for kidney and liver function and urinalysis were normal. Primary immunologic work-up including lymphocyte subset and immunoglobulin levels were normal. Tests for malignancies (tumor markers and peripheral blood smear) and infectious investigations, including *Mantoux Test and IGRA Assay* resulted negative.

Chest X-ray was negative. Magnetic resonance imaging (*MRI)* examination of the superior and inferior abdomen with contrast medium confirmed increased liver dimensions and its structural inhomogeneity with zones of signal alteration: some nodular, other confluent. It also revealed increased spleen size with zones of nodular signal alteration and multiple nodular formations in the following locations: hepatic hilar, mesenteric, lombo-aortic, at the retrocavity of the epiploon, and the greatest one in front of the celiac artery (diameter > 3.5 cm).

**Fig. 1:**
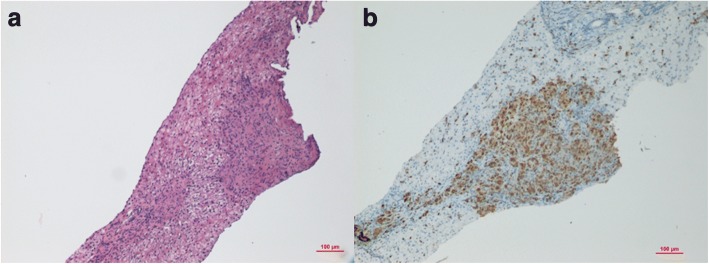
Liver biopsy revealed the presence of small non-caseating granulomas (**a**) consisting of inflammatory epitheliod cells showing positive immunostaining for the monocyte/macrophages marker CD68 (**b**). Original Magnification: 10X

**Fig. 2:**
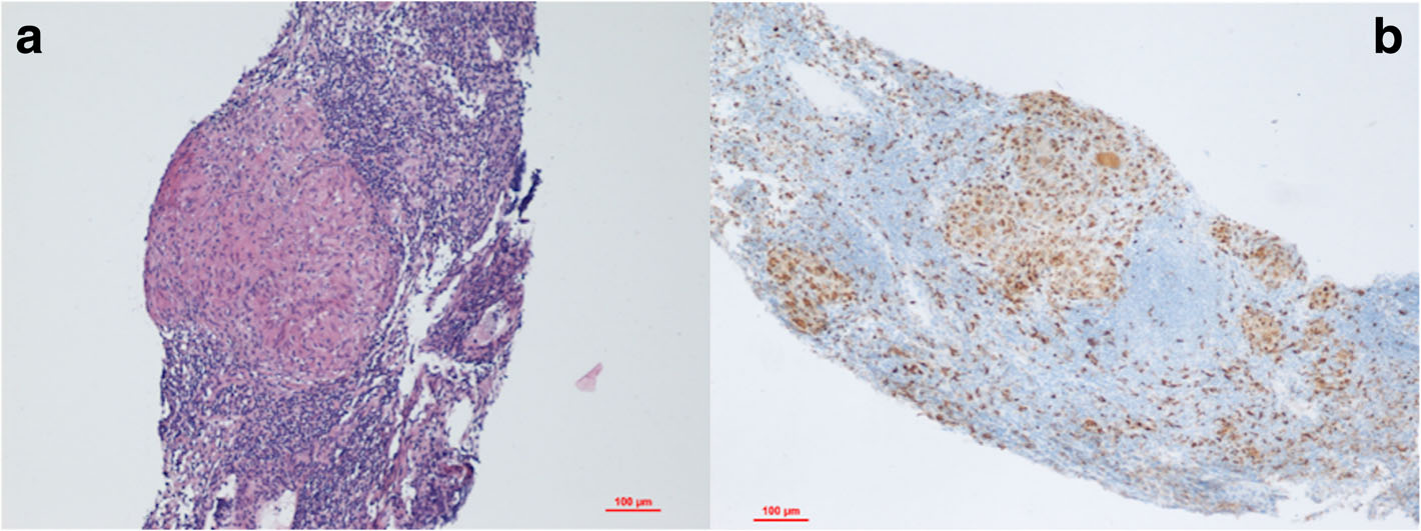
Lymphonode biopsy revealed the presence of small non-caseating granulomas (**a**) consisting of inflammatory epitheliod cells showing positive immunostaining for the monocyte/macrophages marker CD68 (**b**). Original Magnification: 10X

Suspecting a systemic lymphoproliferative disease, we performed liver and lymph node ultrasound-guided biopsy, which showed negativity of Polymerase chain reaction (*PCR)* for potential agents of infectious diseases (including Mycobacteria and Bartonella species) on lymph node material, while it highlighted a non-necrotizing granulomatous inflammation, resembling sarcoidosis, and aspects of non-specific inflammation of the liver. (Fig. 2a, 1a).

Subsequently, we carried out measurements of serum and urinary calcium, serum phosphorus, Angiotensin-Converting Enzyme (ACE), followed by a complete cardiac evaluation, and a complete ophthalmologic evaluation (including slit lamp), all of which resulted within normality.

At this point, given the biopsy results, we performed further lung studies: pulmonary function tests which showed mild restriction and decreased alveolar capillary diffusion. The chest X-Ray revaluation revealed a pattern compatible with mild fibrosis and enlarged lymph nodes. We decided to carry out a high-resolution chest computed tomography (CT), which showed widespread fibrous strands and multiple enlarged lymph nodes (right paratracheal area, at the supraaortic trunk origin, subcarinal, with the largest in this location measuring 30 × 17 mm, pericardiophrenic, bilaterally at axillary level and along the mammary vessels). The bronchoscopic investigation with analysis of the broncho-alveolar lavage (BAL) fluid revealed: macrophages 70%, neutrophils 4% and lymphocytes 26% with a CD4/CD8 ratio of 9.2 (pathological value > 3.5).

Based on these results, we carried out further investigations on liver tissue and lymph node with histochemical techniques: CD68 + nodules were found, suggestive of microgranulomas (Fig. 1b and 2b).

Having ruled out other diagnoses, with suggestive histological findings in two different tissues (liver and lymph nodes) and considering lung involvement, we made the diagnosis of pediatric-onset adult sarcoidosis.

During hospitalization, the condition of the child had always been good and she had always been afebrile. Considering lymph node hypertrophy and the signs of initial portal hypertension (due to compression of the hepatic vessels), we started therapy with prednisone 40 mg/day, and subsequent cross-therapy with mycophenolate mofetil (250 mg/m^2^ increasing up to 1 g/m^2^).

The girl came back to our attention a month after discharge for a follow-up visit: she was found in good general condition, with hepatomegaly and without other clinical signs or symptoms. A brain MRI was performed in order to rule out cerebral involvement and it revealed normal findings. One year later she maintained good clinical condition and normal laboratory tests, therefore MMF was gradually reduced and definitively suspended after 18 months. Now her periodic follow up consist of clinical and laboratory evaluation every 6 months and annual pulmonary function tests, unless clinical or laboratory new findings.

## Discussion and conclusions

Classic sarcoidosis is a rare and poorly described entity in childhood. Clinical manifestations can be significantly variable and non-specific since it is a multisystem disease, and virtually any organ system may be involved. Systemic symptoms as fatigue, fever and weight loss, that in children are usually attributed to infectious, metabolic or lymphoproliferative disorders, are the most common clinical manifestations on presentation, contrary to adults [7].

Cough, dyspnea, crackles and rhonchi at auscultation are the classic clinical features of lung involvement. Chest X-rays result normal in 44% of cases, but high-resolution CT (HRCT) scans reveal abnormalities, mainly nodules with ground-glass opacities and hilo-mediastinal lymphadenopathies, in 95% of children [8]. Restrictive lung disease is the typical alteration found in static and dynamic pulmonary function tests. An elevated CD4/CD8 ratio (≥ 3.5) of the BAL can be a helpful tool in the diagnosis of sarcoidosis, although highly specific but not sensitive [9].

Extra-pulmonary sarcoidosis, mainly associated with a concomitant thoracic involvement, is common and often clinically silent. Liver, spleen, peripheral lymph nodes, skin, eyes, heart and central nervous system are the most commonly involved sites [10].

There is no specific diagnostic test for sarcoidosis, and diagnosis can be considered reliable in presence of suggestive clinical and radiologic manifestations, and histological evidence of non-necrotizing granulomas, when every alternative disease can be reasonably excluded [11, 12].

Our patient presented a subtle disease onset, with general symptoms as asthenia and weight loss. Clinical examination subsequently revealed involvement of liver and spleen, in absence of cutaneous or musculoskeletal signs or sympthoms.

In presence of negative laboratory tests (inflammatory markers, liver function tests, electrolytes, infectious investigations), and chest X-ray, abdominal MRI and biopsy were first oriented to assess a lymphoproliferative disease. Histology in lymph node and liver tissue allowed us to exclude both lymphoma and infectious agents, while it revealed non-caseating granulomas.

Granulomatous disorders comprise a large family sharing the common histological denominator of granuloma formation, in which granuloma acts as a complex inflammatory focus in which organisms or other substances resistant to degradation are circumscribed. Infections are the prevalent underlying aetiology of granulomas: tuberculosis, toxoplasmosis, leishmaniosis, syphilis, brucellosis, fungal (Blastomycosis, cryptococcosis, histoplasmosis), and viral hepatitis (Hepatitis C, CMV, EBV) are the most common causes [13]. Among non-infectious diseases, we can consider neoplasm, adverse drug reactions, and systemic autoimmune disorders; while chronic granulomatous disease of childhood, Kikuchi’s disease and Melkersson-Rosenthal Syndrome are unusual causes [13–15] (Table 1). In the Western world, sarcoidosis and tuberculosis are the two major causes of granulomas [16].

**Table 1:** Main causes of hepatic granulomas

Infectious disease (i.e. HBV, HCV, CMV, Syphilis, Mycobacteria, Bartonella, Brucella, fungal, parasitic)	
Drugs (i.e sulfa drugs, allopurinol, isoniazid, chlorpropamide, quinidine and phenylbutazone)	
Malignancies (i.e Hodgkin and non-Hodgkin lymphoma, metastasis)	
Autoimmune (i.e SLE, polyarteritis nodosa, primary biliary cirrhosis, sclerosing cholangitis, bowel inflammatory disease)	
Chronic granulomatous disease of childhood	
Sarcoidosis	
Foreign body (e.g. talc)	
Lipogranuloma	
Post-operative (i.e. after jejunoileal bypass)	
Idiopathic	

In our patient, once we had ruled out infections, malignancies and granulomatous diseases of childhood, we considered the possibility of sarcoidosis with an apparently exclusive extra-pulmonary expression. Although in the absence of clinical signs and alterations at chest X-ray, pulmonary function tests were considered, since they are easy to perform and non-invasive. They confirmed a restrictive lung disease, promoting further exams (HRCT and BAL), which provided additional data in favor of sarcoidosis.

Long-term studies in children are lacking, but prognosis seems to be similar to adults, with most patients showing spontaneous improvement [17]. However, sarcoidosis remains an enigmatic disease with high variability in extent, severity and long-term outcome. At present there is no curative treatment regimen and, since a large percentage of cases have a self-limiting course (with spontaneous remission in few years), treatment should be administered with caution [15, 18, 19]. Corticosteroids are the first-line therapy; second-line drugs include different alternatives such as methotrexate, TNFα-antagonists, cyclophosphamide, mycophenolate mofetil (MMF) and tacrolimus. [20]

We decided to treat our patient because of an initial compression of portal venules by lymph nodes and the enlargement of chest lymph nodes. Considering her remarkable liver involvement, MMF was than introduced (together with prednisolone) as an adjunctive steroid-sparing treatment [21]. The addition of MMF to corticosteroids is a viable and safe treatment option in sarcoidosis; it allows a significant reduction of maintenance corticosteroids while preserving a stable or even improved clinical condition [22, 23].

The aim of therapy, when pulmonary disease is progressive, is to avoid fibrosis, honeycombing and irreversible lung disease. Absolute indications include progressive stage III disease with symptoms, progressive restriction and specific pulmonary function changes. Several conditions require treatment in order to prevent a poor outcome: posterior or intermediate uveitis; anterior uveitis refractory to topical therapy; disfiguring cutaneous disease (e.g., lupus pernio); cardiac sarcoidosis (when it causes cardiomyopathy, dysrhythmias, or atrioventricular block); brain or spinal cord sarcoidosis (except for isolated VII cranial nerve palsy or mild acute aseptic meningitis); symptomatic hepato-splenic sarcoidosis (i.e. pain, consumptive cytopenia, nausea); and significant hypercalcemia [24, 25].

In conclusion pediatric sarcoidosis is a challenging diagnosis [26], due to lack of specific signs and symptoms. For this reason, general pediatricians should consider sarcoidosis in the differential diagnosis in case of fatigue, fever and weight loss, that represent the most common clinical manifestations on presentation in children, in order to refer promptly the young patient to specialist evaluation [27].

## Abbreviations

ACE: Angiotensin-Converting Enzyme
BAL: Broncho-alveolar lavage
CRP: C-reactive protein
CT: Computed tomography
ESR: Erythrocyte sedimentation rate
Hb: Hemoglobin
HRCT: High-resolution computed tomography
IGRA: Interferon-γ release assays
MCV: Mean corpuscular volume
MMF: Mycophenolate mofetil
MRI: Magnetic resonance imaging
PCR: Polymerase chain reaction
